# 40 years of adding more fructose to high fructose corn syrup than is safe, through the lens of malabsorption and altered gut health–gateways to chronic disease

**DOI:** 10.1186/s12937-024-00919-3

**Published:** 2024-02-02

**Authors:** Luanne Robalo DeChristopher

**Affiliations:** East Fishkill, USA

**Keywords:** High fructose corn syrup, Excess-free-fructose, Fructose, Malabsorption, Dysbiosis, Microbiome, Apple juice, Asthma, Fructositis, Glycation, Fructosylation

## Abstract

Labels do not disclose the excess-free-fructose/unpaired-fructose content in foods/beverages. Objective was to estimate excess-free-fructose intake using USDA loss-adjusted-food-availability (LAFA) data (1970–2019) for high fructose corn syrup (HFCS) and apple juice, major sources of excess-free-fructose, for comparison with malabsorption dosages (~ 5 g-children/ ~ 10 g-adults). Unlike sucrose and equimolar fructose/glucose, unpaired-fructose triggers fructose malabsorption and its health consequences. Daily intakes were calculated for HFCS that is generally-recognized-as-safe/ (55% fructose/45% glucose), and variants (65/35, 60/40) with higher fructose-to-glucose ratios (1.9:1, 1.5:1), as measured by independent laboratories. Estimations include consumer-level-loss (CLL) allowances used before (20%), and after, subjective, retroactively-applied increases (34%), as recommended by corn-refiners (~ 2012). No contributions from crystalline-fructose or agave syrup were included due to lack of LAFA data. High-excess-free-fructose-fruits (apples/pears/watermelons/mangoes) were not included. Eaten in moderation they are less likely to trigger malabsorption. Another objective was to identify potential parallel trends between excess-free-fructose intake and the “unexplained” US asthma epidemic. The fructose/gut-dysbiosis/lung axis is well documented, case-study evidence and epidemiological research link HFCS/apple juice intake with asthma, and unlike gut-dysbiosis/gut-fructosylation, childhood asthma prevalence data spans > 40 years.

**Results**

Excess-free-fructose daily intake for individuals consuming HFCS with an average 1.5:1 fructose-to-glucose ratio, ranged from 0.10 g/d in 1970, to 11.3 g/d in 1999, to 6.5 g/d in 2019, and for those consuming HFCS with an average 1.9:1 ratio, intakes ranged from 0.13 g/d to 16.9 g/d (1999), to 9.7 g/d in 2019**,** based upon estimates with a 20% CLL allowance. Intake exceeded dosages that trigger malabsorption (~ 5 g) around ~ 1980. By the early 1980’s, tripled apple juice intake had added ~ 0.5 g to average-per-capita excess-free-fructose intake. Contributions were higher (~ 3.8 g /4-oz.) for individuals consuming apple juice consistent with a healthy eating pattern (4-oz. children, 8-oz. adults). The “unexplained” childhood asthma epidemic (1980-present) parallels increasing average-per-capita HFCS/apple juice intake trends and reflects epidemiological research findings.

**Conclusion**

Displacement of sucrose with HFCS, its ubiquitous presence in the US food-supply, the industry practice of adding more fructose to HFCS than generally-recognized-as-safe, and increased use of apple juice/crystalline fructose/agave syrup in foods/beverages has contributed to unprecedented excess-free-fructose intake levels, fructose malabsorption, gut-dysbiosis and gut-fructosylation (immunogen burden)-gateways to chronic disease.

## Background

HFCS [1, 2] and apple juice [3] are significant sources of excess-free-fructose [1–3]–the fructose type that occurs when the fructose-to-glucose ratio exceeds 1:1, i.e. unpaired fructose. Excess-free-fructose, also known as unpaired fructose, triggers fructose malabsorption [4–17] – a condition associated with gut dysbiosis and the gut/lung, gut/heart, gut/brain, and gut/kidney axes [17–25], and a wide range of adverse health consequences (asthma, COPD, autoimmune disease, inflammatory bowel disease (IBD) / syndrome (IBS), cardiovascular disease (CVD), non-alcohol associated fatty liver disease (NAFLD), chronic kidney disease (CKD), and mental health and cardiometabolic disorders) [17–50]; excess-free-fructose forms gut immunogens (advanced glycation end-products (FruAGE)) via glycation / fructosylation of dietary peptides and incretins with far reaching consequences [26–30]. FruAGE bind receptors (RAGE) that are central mediators of asthma [51, 52]. Sucrose, a disaccharide of fructose and glucose, is not associated with fructose malabsorption, nor are equimolar concentrations of fructose and glucose [6], i.e., when in a ~ 1:1 fructose-to-glucose ratio [4–17].

Mechanisms that drive differences in unpaired fructose absorption capacity are still being explored. Researchers have determined that lower expression of ChREBP, a GLUT5 regulatory gene, likely underlies fructose malabsorption [53, 54]. In murine research, investigators found that gene deletion of ChREBP, which is expressed in the proximal gut epithelium where carbohydrate digestion and absorption primarily occur and in GLP-1 producing L cells, resulted in impaired expression of glucose, galactose, and fructose transporters, and was accompanied by severe fructose malabsorption syndrome and reduced production of GLP-1 – a hormone that stimulates pancreatic β cells to produce insulin and regulates satiety [54, 55]. Different alleles and/or variability in ChREBP gene expression may explain differences in excess-free-fructose absorption capacity across individuals. Children are more likely to experience fructose malabsorption at lower intakes [14–16], and limited research shows African Americans have higher fructose malabsorption prevalence than other groups [55].

The fructose fraction in HFCS has been higher than thought. Research by the Keck School of Medicine showed that the fructose-to-glucose ratio in the HFCS in popular beverages has been higher (1.9:1 [1] & 1.5:1 [2]) than generally-recognized-as-safe (1.2:1) (GRAS) [56]. Therefore, consumers have been unwittingly eating more unpaired fructose (excess-free-fructose) than is considered safe. The shift from sucrose to HFCS in US soft drinks (~ 1984) [57–63], its proliferation in the US food supply (~ 1980—present) [57–63], and industry practice of using higher fructose-to-glucose ratios (1.9:1 [1], 1.5:1 [2]) than GRAS, has occurred at the expense of exposing US consumers to unsafe excess-free-fructose levels [57–67], in order to achieve higher sweetness at lower cost [64, 65]. Less is needed as fructose is ~ twice as sweet as glucose [17]. Consumers seeking to limit excess-free-fructose consumption are hampered by the fact that US food labels *do not* provide information on the fructose or excess-free-fructose content in foods and beverages. The industry practice of adding more fructose to HFCS than is generally-recognized-as-safe has contributed to underestimations of total fructose and excess-free-fructose intake. Importantly, average per capita excess-free-fructose intake estimates are lacking.

## Study objectives

The first objective of this analysis is to estimate average per capita daily excess-free-fructose intake from HFCS and apple juice, major sources of excess-free-fructose in the American diet, by utilizing USDA loss adjusted food availability (LAFA) data for the years for which data is available (1970 – 2019) [62–65]. The US Department of Agriculture (USDA) publishes LAFA data for a broad array of foods including added sweeteners and 100% fruit juices. The USDA “utilizes this data series to estimate *average per capita daily intake of foods and sweeteners over time*” [62]. Source data used in this analysis can be found at https://www.ers.usda.gov/data-products/food-availability-per-capita-data-system/.

Other growing and increasingly popular sources of excess-free-fructose (EFF), i.e. crystalline fructose (nearly 100% fructose) and agave syrup (70 – 90% fructose) [68], are *not* included in this analysis, as *individual* LAFA data for these high excess-free-fructose sweeteners are not available/published by the USDA. Consumption trends for high excess-free-fructose fruits (apples, pears, watermelons and mangoes) [3] are also *not included herein* because when eaten in moderation, these fruits are less likely to trigger fructose malabsorption and its health consequences.

Apple juice is included as it contains a high fructose-to-glucose ratio (~ 2.1:1) [3], higher than measured in HFCS (1.5:1 and 1.9:1) by independent labs [1, 2]. Apple juice is the second most consumed juice after orange juice [62, 69]. Intake has tripled since 1970 [62, 69]. Therefore, it is a significant source of excess-free-fructose (~ 8 g per 250 ml) [3]. Children ≤ 5 years of age are major consumers [70, 71] and they are fructose malabsorbers at lower excess-free-fructose (EFF) intakes [14–16]. Orange and grape juices are not included herein, as they contain negligible amounts of excess-free-fructose (orange juice (~ 0.4 g EFF, per 250 ml) / grape juice (~ 2 g EFF, per 250 ml)) [3]. Given their low EFF concentrations, they are negligible contributors of excess-free-fructose.

The second objective of this analysis is to assess the impact of adding more fructose to HFCS than is generally-recognized-as-safe [56] on excess-free-fructose consumption trends. Hence, excess-free-fructose average per capita intake estimations are derived for high fructose HFCS variants (65% fructose & 35% glucose; 60% fructose & 40% glucose), as measured in popular beverages, by independent laboratories [1, 2]. Notably, beverages are the most significant source of HFCS in the American diet – by a wide margin [57]. Estimations herein *do not* include HFCS 42, as it is reasonable to assume, given independent lab results of the HFCS in beverages, that baked goods contain more fructose than assumed, and likely follow a similar practice as measured in beverages.

Excess-free-fructose intake estimates derived herein, will enable comparisons with dosages known to trigger malabsorption and its health consequences (~ 5 g pediatric/ and ~ 10 g adult) [4–17]. Excess-free-fructose daily intakes are also calculated for HFCS when combined with apple juice intake consistent with a healthy eating pattern [72] for children (124 g (*1/2 cup*)), and adults (248 g (*1 cup*)).

The third objective of this analysis is to identify potential parallel trends between excess-free-fructose intake trends and the “unexplained” US childhood asthma epidemic – “a disease condition linked to unpaired fructose induced gut dysbiosis [17–22] and the gut/lung axis [23–25];” and to elevated receptor of advanced glycation end-product (RAGE) signaling [51, 52]. The fructose/gut dysbiosis/lung axis is well documented [17–25], and research confirms that the gut is a source of asthma triggering immunogens (AGE / FruAGE) formed via fructosylation of peptides (partially digested dietary proteins and incretins) [27–30]. Clinical studies demonstrate that increased RAGE ligands and signaling strongly correlate with asthma severity [51]. Receptors of AGE / FruAGE [51, 52] (immunogens that form in the gut with unabsorbed unpaired fructose) [26–30] are mediators of asthma [31–33].

Case study evidence [26] and epidemiological research link high fructose corn syrup intake with asthma [31–46], and unlike gut dysbiosis, childhood asthma prevalence data spans > 40 years, i.e., before and after the advent of HFCS in the US food supply. Thus, childhood asthma prevalence [73–75] is plotted herein with intake trends for HFCS and apple juice. A comparative plot includes intake trends for orange juice—a 100% juice with comparable total fructose and glycemic load (11 g per 250 ml/ 15 glycemic units) as apple juice (15.7 g per 250 ml/ 12 glycemic units), but low excess-free-fructose (0.4 g per 250 ml) [3]. Future analyses should explore potential parallel trends with cardiometabolic and chronic kidney disease, i.e. diseases with available prevalence data and consistent epidemiological research that links EFF intake with increased risks and mortality.

## Methods

Microsoft Excel, and RStudio Version 1.4.1106 were used to calculate and plot average per capita excess-free-fructose daily intakes from HFCS and apple juice, based upon USDA loss adjusted (retail and consumer level) food availability data, for the time period between 1970 and 2019 [62–64]. Three intake estimates have been calculated for HFCS. One estimate is based on HFCS with a 1.5:1 fructose-to-glucose ratio (60% fructose / 40% glucose), as measured by independent laboratories in 2014 [2]; the second estimate is based on HFCS with a 1.9:1 fructose-to-glucose ratio (65% fructose / 35% glucose), as measured by independent laboratories in 2010 [1]; and another is based on HFCS with a 1.2:1 fructose-to-glucose ratio (55% fructose / 45% glucose), i.e. the ratio that is generally-recognized-as-safe [56].

For this analysis, retail loss (11%) adjusted, average per capita HFCS availability data have been converted to grams per day for each year (g/d/y) herein (1970 – 2019) (lb./yr * 453.59)/365) (Tables 1, 2 and 3). Annual retail loss adjusted data (g/d/y) were then updated to include consumer level loss (CLL) allowances which account for “cooking loss and uneaten food” [64, 65]. However, due to controversy surrounding the accuracy of USDA’s consumer level loss allowance for HFCS [63–65], three intake estimates have been calculated for each HFCS variant. One estimate includes a 15% consumer level loss factor, as recommended by independent researchers tasked with improving CLL accuracy (2012) [63]; another estimate includes the historically used CLL factor (20%); and the third estimate includes the newly adopted (2012), *retroactively applied* CLL factor (34%), as recommended for HFCS by US corn refiners – a biased group [63–65].

**Table 1:** Excess-free-fructose intake (g/d) from HFCS that is 55% fructose extrapolated from average per capita loss adjusted food availability (LAFA) data^a^

**Year**	**Unadjusted per capita availability of HFCS in lb/y**^a^	**Per capita availability of HFCS (lbs/y) after retail loss of 11%**^a^	**Per capita availability of HFCS (g/d) after retail loss of 11%** g/d = ((lbs/yr * 453.59)/365)	**Excess-free-fructose (g/d) after 11% retail and 15% consumer level loss allowance**	**Excess-free-fructose (g/d) after 11% retail and 20% consumer level loss allowance**	**Excess-free-fructose (g/d) after 11% retail and 34% consumer level loss allowance**
1970	0.5	0.4	0.6	0.05	0.04	0.04
1971	0.8	0.7	0.9	0.08	0.16	0.06
1972	1.2	1.1	1.3	0.11	0.11	0.09
1973	2.1	1.9	2.3	0.20	0.19	0.15
1974	2.8	2.5	3.1	0.26	0.25	0.20
1975	4.9	4.3	5.4	0.46	0.43	0.36
1976	7.2	6.4	7.9	0.68	0.64	0.53
1977	9.6	8.5	10.6	0.90	0.85	0.70
1978	10.8	9.6	11.9	1.02	0.95	0.79
1979	14.8	13.1	16.3	1.39	1.30	1.08
1980	19	16.9	21.0	1.79	1.68	1.39
1981	22.8	20.3	25.3	2.14	2.02	1.66
1982	26.6	23.7	29.4	2.50	2.35	1.94
1983	31.2	27.8	34.5	2.93	2.76	2.28
1984	37.2	33.1	41.2	3.50	3.29	2.72
1985	45.2	40.2	50.0	4.25	4.00	3.30
1986	45.7	40.7	50.5	4.30	4.04	3.34
1987	47.7	42.5	52.8	4.48	4.22	3.48
1988	49.0	43.6	54.2	4.61	4.34	3.58
1989	48.2	42.9	53.3	4.53	4.26	3.52
1990	49.6	44.1	54.9	4.66	4.39	3.62
1991	50.3	44.8	55.6	4.73	4.45	3.67
1992	51.8	46.1	57.4	4.87	4.58	3.78
1993	54.5	48.5	60.3	5.12	4.82	3.98
1994	56.2	50.0	62.2	5.28	4.97	4.10
1995	57.6	51.3	63.8	5.42	5.10	4.20
1996	57.4	51.1	63.6	5.40	5.08	4.19
1997	60.7	54.0	67.1	5.71	5.37	4.43
1998	62.0	55.2	68.7	5.83	5.49	4.53
1999	63.8	56.7	70.6	6.00	5.65	4.66
2000	62.5	55.6	69.2	5.88	5.53	4.56
2001	62.2	55.3	68.8	5.85	5.50	4.54
2002	62.5	55.6	69.2	5.88	5.53	4.56
2003	60.5	53.8	67.0	5.69	5.35	4.42
2004	59.5	53.0	66.0	5.59	5.26	4.34
2005	58.8	52.3	65.1	5.53	5.20	4.29
2006	57.8	51.5	64.0	5.43	5.11	4.22
2007	55.8	49.7	61.8	5.25	4.94	4.07
2008	52.6	46.8	58.3	4.95	4.65	3.84
2009	49.6	44.2	54.9	4.66	4.39	3.62
2010	48.3	43.0	53.5	4.54	4.27	3.53
2011	46.7	41.5	51.7	4.39	4.13	3.41
2012	45.7	40.7	50.6	4.30	4.04	3.34
2013	43.7	38.9	48.3	4.11	3.87	3.19
2014	43.4	38.6	48.3	4.08	3.84	3.17
2015	42.5	37.8	47.0	4.00	3.76	3.10
2016	41.4	36.8	45.7	3.89	3.66	3.02
2017	39.8	35.4	44.0	3.74	3.52	2.91
2018	37.7	33.5	41.6	3.54	3.33	2.75
2019	36.7	32.7	40.6	3.45	3.25	2.68

^a^Source is the U.S. Department of Agriculture, Economic Research Service (ERS). The ERS Food Availability (Per Capita) Data System. Economic Research Service Home Page, https://www.ers.usda.gov/data-products/food-availability-per-capita-data-system/

https://www.ers.usda.gov/data-products/food-availability-per-capita-data-system/food-availability-documentation/

**Table 2:** Excess-free-fructose intake (g/d) from HFCS that is 60% fructose, as extrapolated from average per capita loss adjusted food availability (LAFA) data^a^

**Year**	**Unadjusted per capita availability of HFCS in lb/y**^a^	**Per capita availability of HFCS (lb/y) after retail loss of 11%**^a^	**Per capita availability of HFCS (g/d) after retail loss of 11%** g/d = ((lbs/yr * 453.59)/365)	**Excess-free-fructose (g/d) after 11% retail and 15% consumer level loss allowance**	**Excess-free-fructose (g/d) after 11% retail and 20% consumer level loss allowance**	**Excess-free-fructose (g/d) after 11% retail and 34% consumer level loss allowance**
1970	1.5	1.4	0.6	0.10	0.10	0.07
1971	0.8	0.7	0.9	0.15	0.14	0.12
1972	1.2	1.1	1.3	0.23	0.21	0.17
1973	2.1	1.9	2.3	0.39	0.37	0.31
1974	2.8	2.5	3.1	0.53	0.50	0.41
1975	4.9	4.3	5.3	0.9	0.9	0.7
1976	7.2	6.4	8.0	1.4	1.3	1.1
1977	9.6	8.5	10.6	1.8	1.7	1.4
1978	10.8	9.6	11.9	2.0	1.9	1.6
1979	14.8	13.1	16.3	2.8	2.6	2.2
1980	19.0	16.9	21.0	3.6	3.4	2.8
1981	22.8	20.3	25.2	4.3	4.0	3.3
1982	26.6	23.7	29.5	5.0	4.7	3.9
1983	31.2	27.8	34.5	5.9	5.5	4.6
1984	37.2	33.1	41.1	7.0	6.6	5.4
1985	45.2	40.2	50.0	8.5	8.0	6.6
1986	45.7	40.7	50.6	8.6	8.1	6.7
1987	47.7	42.5	52.8	9.0	8.4	7.0
1988	49.0	43.6	54.2	9.2	8.7	7.2
1989	48.2	42.9	53.3	9.1	8.5	7.0
1990	49.6	44.1	54.8	9.3	8.8	7.2
1991	50.3	44.8	55.7	9.5	8.9	7.3
1992	51.8	46.1	57.3	9.7	9.2	7.6
1993	54.5	48.5	60.3	10.2	9.6	8.0
1994	56.2	50.0	62.1	10.6	9.9	8.2
1995	57.6	51.3	63.8	10.8	10.2	8.4
1996	57.4	51.1	63.5	10.8	10.2	8.4
1997	60.7	54.0	67.1	11.4	10.7	8.9
1998	62.0	55.2	68.6	11.7	11.0	9.1
1999	63.8	56.7	70.5	12.0	11.3	9.3
2000	62.5	55.6	69.1	11.8	11.1	9.1
2001	62.2	55.3	68.7	11.7	11.0	9.1
2002	62.5	55.6	69.1	11.7	11.1	9.1
2003	60.5	53.8	66.9	11.4	10.7	8.8
2004	59.5	53.0	65.9	11.2	10.5	8.7
2005	58.8	52.3	65.0	11.1	10.4	8.6
2006	57.8	51.5	64.0	10.9	10.2	8.4
2007	55.8	49.7	61.8	10.5	9.9	8.1
2008	52.6	46.8	58.2	9.9	9.3	7.7
2009	49.6	44.2	54.9	9.3	8.8	7.2
2010	48.3	43.0	53.4	9.1	8.5	7.1
2011	46.7	41.5	51.6	8.8	8.3	6.8
2012	45.7	40.7	50.6	8.6	8.1	6.7
2013	43.7	38.9	48.3	8.2	7.7	6.4
2014	43.4	38.6	48.0	8.2	7.7	6.3
2015	42.5	37.8	47.0	8.0	7.5	6.2
2016	41.4	36.8	45.7	7.8	7.3	6.0
2017	39.8	35.4	44.0	7.5	7.0	5.8
2018	37.7	33.5	41.7	7.1	6.7	5.5
2019	36.7	32.7	40.6	6.9	6.5	5.4

^a^Source is the U.S. Department of Agriculture, Economic Research Service (ERS). The ERS Food Availability (Per Capita) Data System. Economic Research Service Home Page, https://www.ers.usda.gov/data-products/food-availability-per-capita-data-system/

https://www.ers.usda.gov/data-products/food-availability-per-capita-data-system/food-availability-documentation/

**Table 3:** Excess-free-fructose intake (g/d) from HFCS that is 65% fructose, as extrapolated from average per capita loss adjusted food availability (LAFA) data^a^

**Year**	**Unadjusted per capita availability of HFCS (lb/yr)**^a^	**Per capita availability of HFCS (lb/y) after retail loss of 11%**^a^	Per capita availability of HFCS (g/d) after retail loss of 11% g/d = ((lbs/yr * 453.59)/365)	**Excess-free-fructose (g/d) after 11% retail and 15% consumer level losses**	**Excess-free-fructose (g/d) after 11% retail and 20% consumer level losses**	**Excess-free-fructose (g/d) after 11% retail and 34% consumer level losses**
1970	0.5	0.4	0.6	0.14	0.13	0.11
1971	0.8	0.7	0.9	0.23	0.21	0.18
1972	1.2	1.1	1.3	0.34	0.32	0.26
1973	2.1	1.9	2.3	0.59	0.56	0.46
1974	2.8	2.5	3.1	0.79	0.74	0.61
1975	4.9	4.3	5.3	1.2	1.1	1.1
1976	7.2	6.4	8.0	1.8	1.7	1.6
1977	9.6	8.5	10.6	2.4	2.3	2.1
1978	10.8	9.6	11.9	2.7	2.5	2.4
1979	14.8	13.1	16.3	3.7	3.5	3.2
1980	19.0	16.9	21.0	5.4	5.0	4.2
1981	22.8	20.3	25.2	6.4	6.1	5.0
1982	26.6	23.7	29.5	7.5	7.1	5.8
1983	31.2	27.8	34.5	8.8	8.3	6.8
1984	37.2	33.1	41.1	10.5	9.9	8.1
1985	45.2	40.2	50.0	12.7	12.0	9.9
1986	45.7	40.7	50.6	12.9	12.1	10.0
1987	47.7	42.5	52.8	13.5	12.7	10.4
1988	49.0	43.6	54.2	13.8	13.0	10.7
1989	48.2	42.9	53.3	13.6	12.8	10.6
1990	49.6	44.1	54.8	14.0	13.2	10.9
1991	50.3	44.8	55.7	14.2	13.4	11.0
1992	51.8	46.1	57.3	14.6	13.8	11.3
1993	54.5	48.5	60.3	15.4	14.5	11.9
1994	56.2	50.0	62.1	15.9	14.9	12.3
1995	57.6	51.3	63.8	16.2	15.3	12.6
1996	57.4	51.1	63.5	16.2	15.2	12.6
1997	60.7	54.0	67.1	17.1	16.1	13.3
1998	62.0	55.2	68.6	17.5	16.5	13.6
1999	63.8	56.7	70.5	18.0	16.9	14.0
2000	62.5	55.6	69.1	17.6	16.6	13.7
2001	62.2	55.3	68.7	17.5	16.5	13.6
2002	62.5	55.6	69.1	17.6	16.6	13.7
2003	60.5	53.8	66.9	17.1	16.1	13.2
2004	59.5	53.0	65.9	16.8	15.8	13.0
2005	58.8	52.3	65.0	16.6	15.6	12.9
2006	57.8	51.5	64.0	16.3	15.3	12.7
2007	55.8	49.7	61.8	15.7	14.8	12.2
2008	52.6	46.8	58.2	14.8	14.0	11.5
2009	49.6	44.2	54.9	14.0	13.2	10.9
2010	48.3	43.0	53.4	13.6	12.8	10.6
2011	46.7	41.5	51.6	13.2	12.4	10.2
2012	45.7	40.7	50.6	12.9	12.1	10.0
2013	43.7	38.9	48.3	12.3	11.6	9.6
2014	43.4	38.6	48.0	12.2	11.5	9.5
2015	42.5	37.8	47.0	12.0	11.3	9.3
2016	41.4	36.8	45.7	11.7	11.0	9.1
2017	39.8	35.4	44.0	11.2	10.6	8.7
2018	37.7	33.5	41.7	10.6	10.0	8.2
2019	36.7	32.7	40.6	10.3	9.7	8.0

^a^Source is the U.S. Department of Agriculture, Economic Research Service (ERS). The ERS Food Availability (Per Capita) Data System. Economic Research Service Home Page, https://www.ers.usda.gov/data-products/food-availability-per-capita-data-system/

https://www.ers.usda.gov/data-products/food-availability-per-capita-data-system/food-availability-documentation/

**Fig. 1:**
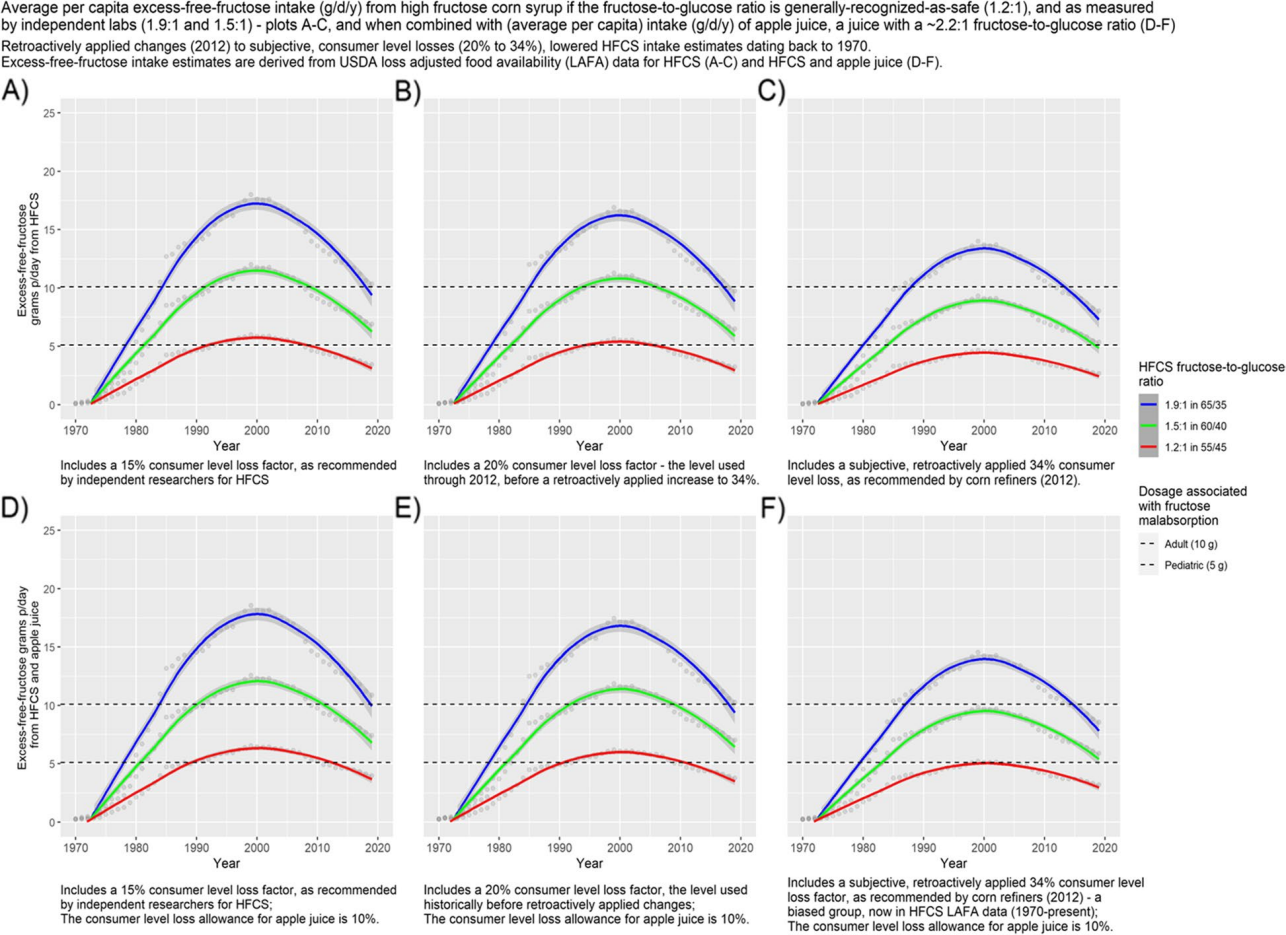
Average per capita excess-free-fructose intake (g/d/y) from high fructose corn syrup if the fructose-to-glucose ratio is generally-recognized-as-safe (1.2:1), and as measured by independent labs (1.9:1 and 1.5:1)—plots **A**-**C**, and when combined with (average per capita) intake (g/d/y) of apple juice, a juice with a ~ 2.2:1 fructose-to-glucose ratio (**D**-**F**). Retroactively applied changes (2012) to subjective, consumer level losses (20% to 34%), lowered HFCS intake estimates dating back to 1970. Excess-free-fructose intake estimates are derived from USDA loss adjusted food availability (LAFA) data for HFCS (**A**-**C**) and HFCS and apple juice (**D**-**F**)

**Fig. 2:**
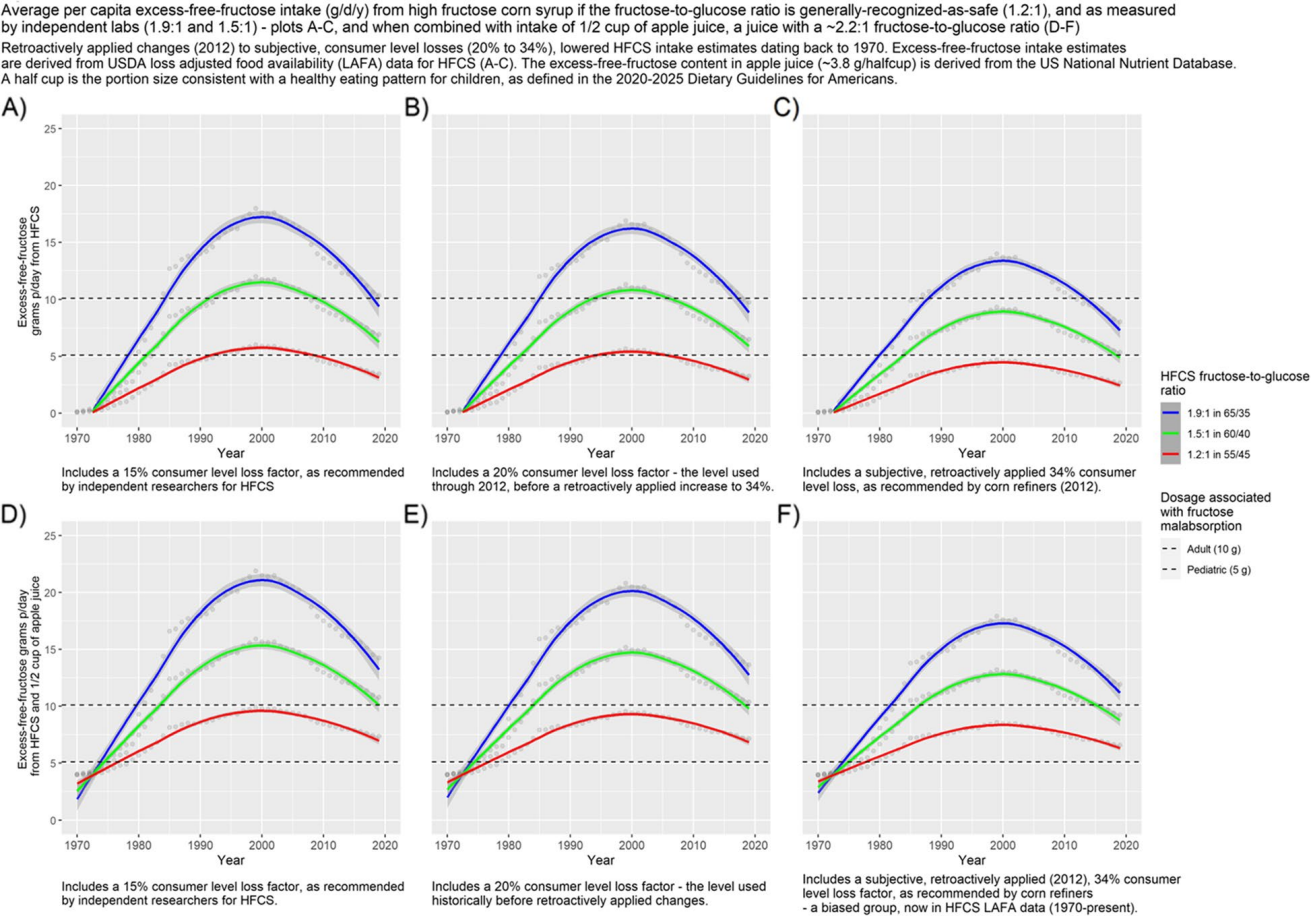
Average per capita excess-free-fructose intake (g/d/y) from high fructose corn syrup if the fructose-to-glucose ratio is generally-recognized-as-safe (1.2:1), and as measured by independent labs (1.9:1 and 1.5:1)—plots **A**-**C**, and when combined with intake of 1/2 cup of apple juice, a juice with a ~ 2.2:1 fructose-to-glucose ratio (**D**-**F**). Retroactively applied changes (2012) to subjective, consumer level losses (20% to 34%), lowered HFCS intake estimates dating back to 1970. Excess-free-fructose intake estimates are derived from USDA loss adjusted food availability (LAFA) data for HFCS (**A**-**C**). The excess-free-fructose content in apple juice (~ 3.8 g/halfcup) is derived from the US National Nutrient Database. A half cup is the portion size consistent with a healthy eating pattern for children, as defined in the 2020–2025 Dietary Guidelines for Americans

**Fig. 3:**
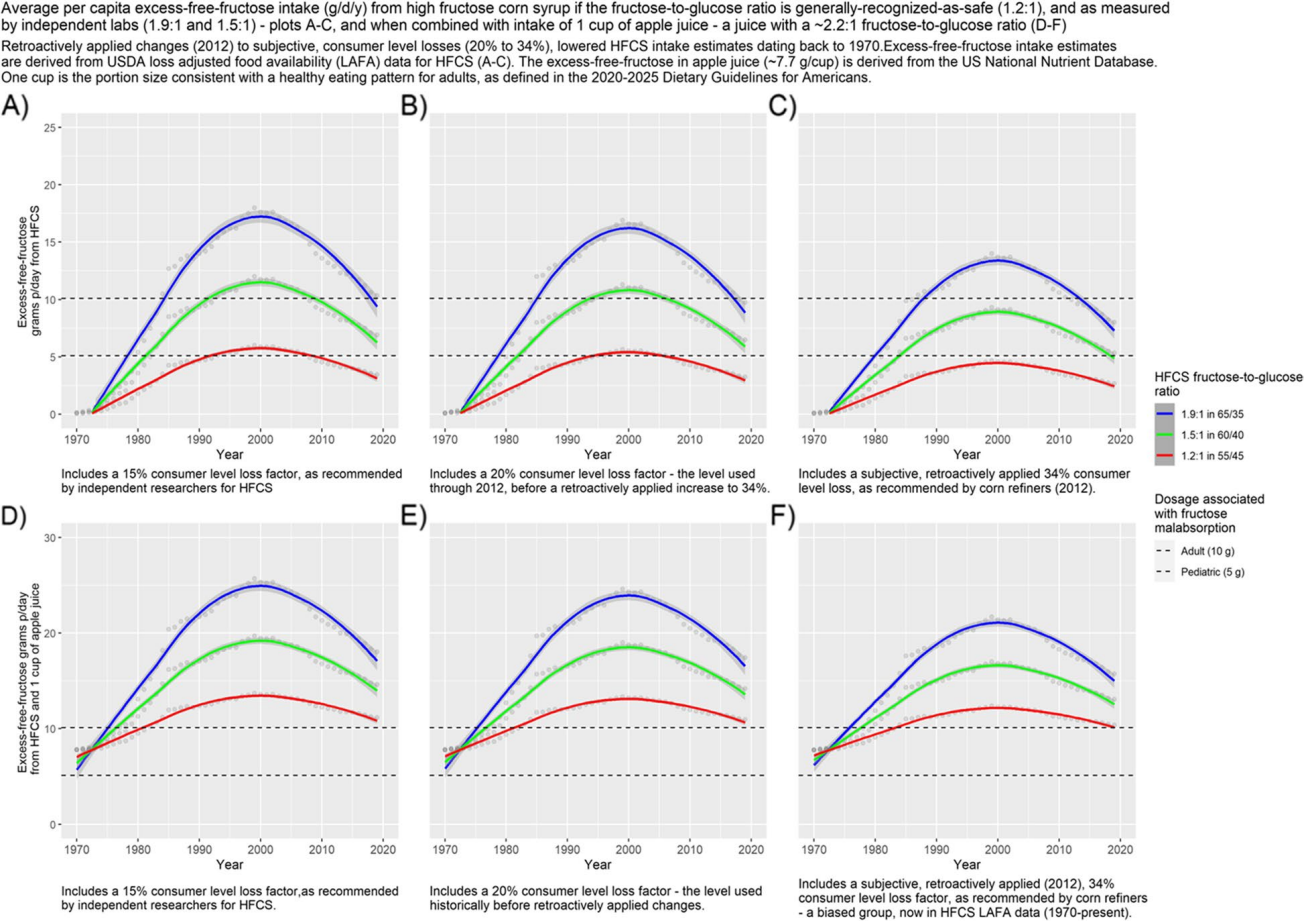
Average per capita excess-free-fructose intake (g/d/y) from high fructose corn syrup if the fructose-to-glucose ratio is generally-recognized-as-safe (1.2:1), and as measured by independent labs (1.9:1 and 1.5:1)—plots **A**-**C**, and when combined with intake of 1 cup of apple juice—a juice with a ~ 2.2:1 fructose-to-glucose ratio (**D**-**F**). Retroactively applied changes (2012) to subj;ective, consumer level losses (20% to 34%), lowered HFCS intake estimates dating back to 1970.Excess-free-fructose intake estimates are derived from USDA loss adjusted food availability (LAFA) data for HFCS (**A**-**C**). The excess-free-fructose in apple juice (-7.7 glcup) is derived from the US National Nutrient Database. One cup is the portion size consistent with a healthy eating pattern for adults, as defined in the 2020–2025 Dietary Guidelines for Americans

The following formulae define the three steps used to derive excess-free-fructose daily intake from HFCS availability data: Step 1 – Subtract the consumer level loss allowance (15% or 20% or 34%) from retail-loss-adjusted data. Step 2 – Calculate the fructose and glucose components in the results from “Step 1” for each HFCS variant (65/35; 60/40; 55/45), i.e. (All-loss-adjusted avg/cap/HFCS avail/g/d/y * the fructose %; All-loss-adjusted avg/cap/HFCS avail/g/d/y * the glucose %). Step 3—Subtract the glucose from the fructose fraction. Results reflect average per capita excess-free-fructose daily intake (g/d/y) and are depicted in Figs. 1, 2 and 3, plots A-C.

The same approach was used to calculate excess-free-fructose daily intake from average per capita LAFA data for apple juice. The fructose to glucose ratio (~ 2.1:1) in apple juice (68/32) was obtained from the US National Nutrients Database (NDB) [3] (No. 09400). 100 g of apple juice contains 5.7 g of fructose + 2.6 g of glucose (1 oz. contains 1.8 g fructose and 0.8 g glucose) [3]. Estimations include USDA’s retail (11%) and consumer level (10%) loss allowances for apple juice. Results are depicted in Fig. 1, plots D – F.

Figures 2 and 3, plots D-F, show contributions to excess-free-fructose intake from half a cup (~ 4 g) and a cup of apple juice (~ 8 g), respectively, i.e. servings consistent with a healthy eating pattern for children and adults [72], as defined in the 2020–2025 Dietary Guidelines for Americans.

**Fig. 4:**
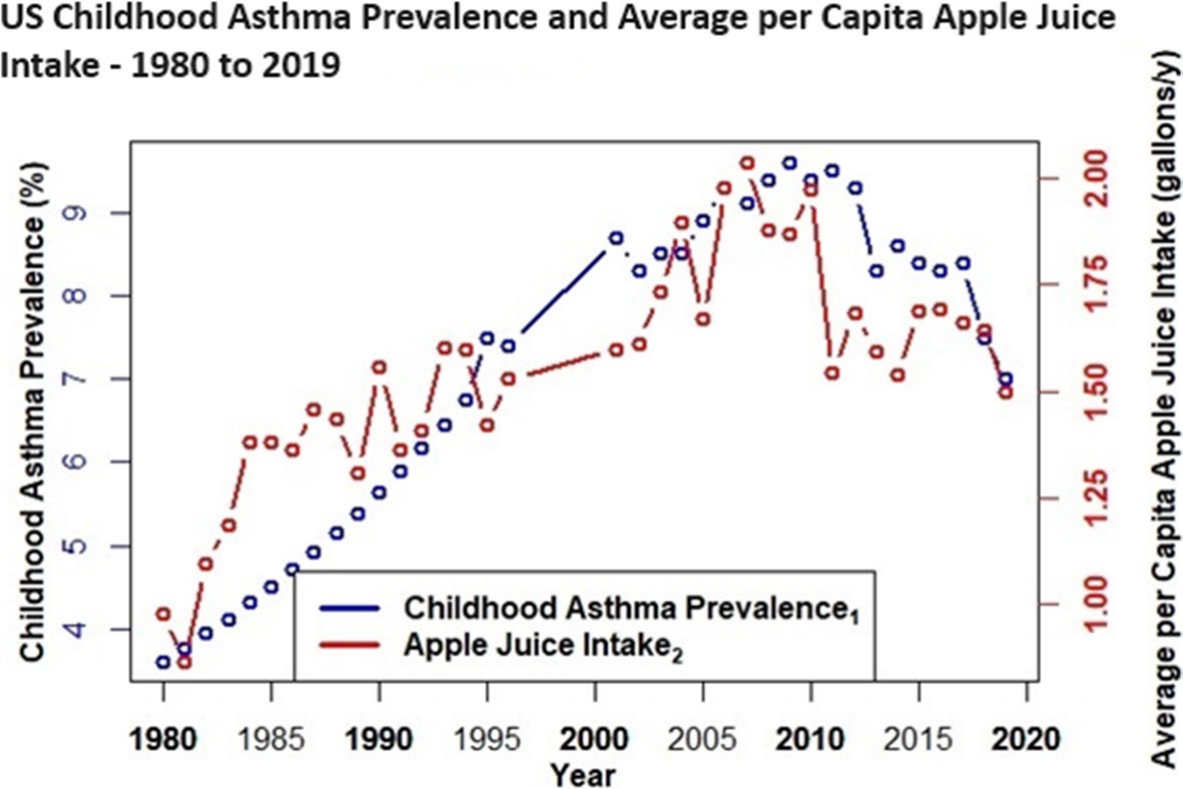
US Childhood Asthma Prevalence and Average per Capita Apple Juice Intake – 1980 to 2019

**Fig. 5:**
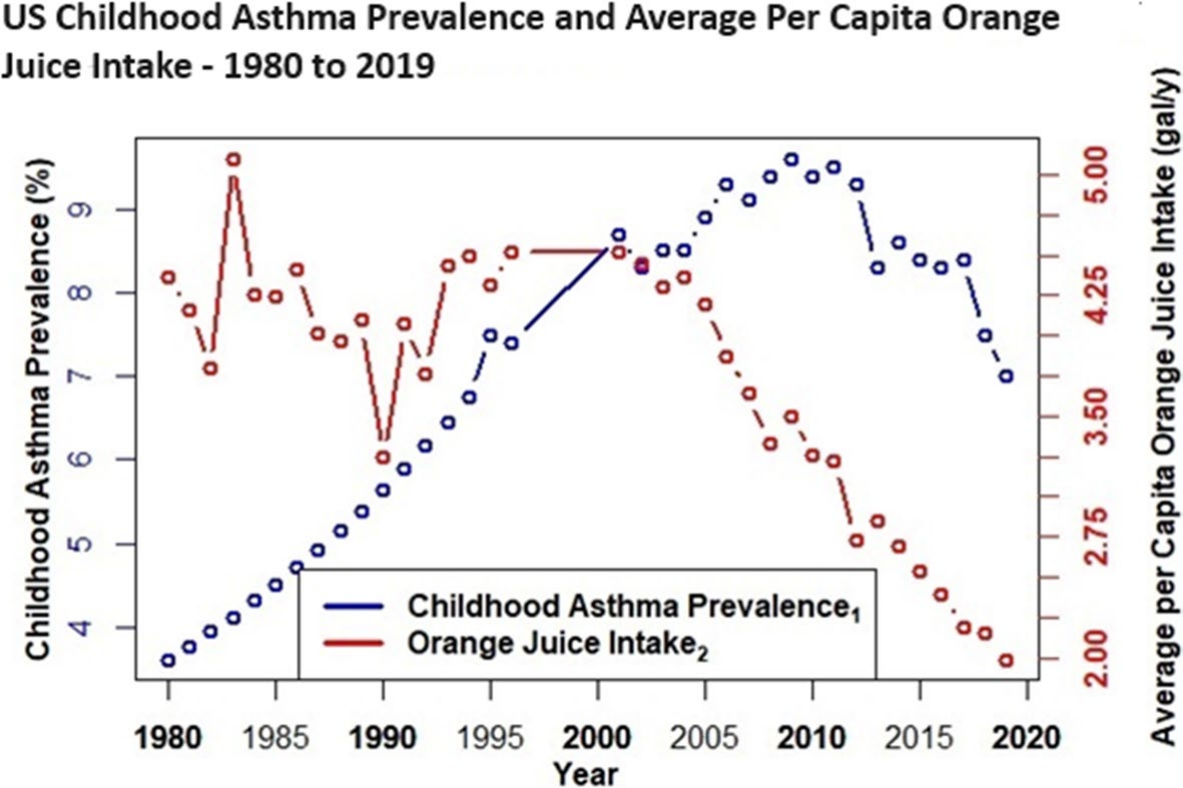
US Childhood Asthma Prevalence and Average Per Capita Orange Juice Intake – 1980 to 2019

**Fig. 6:**
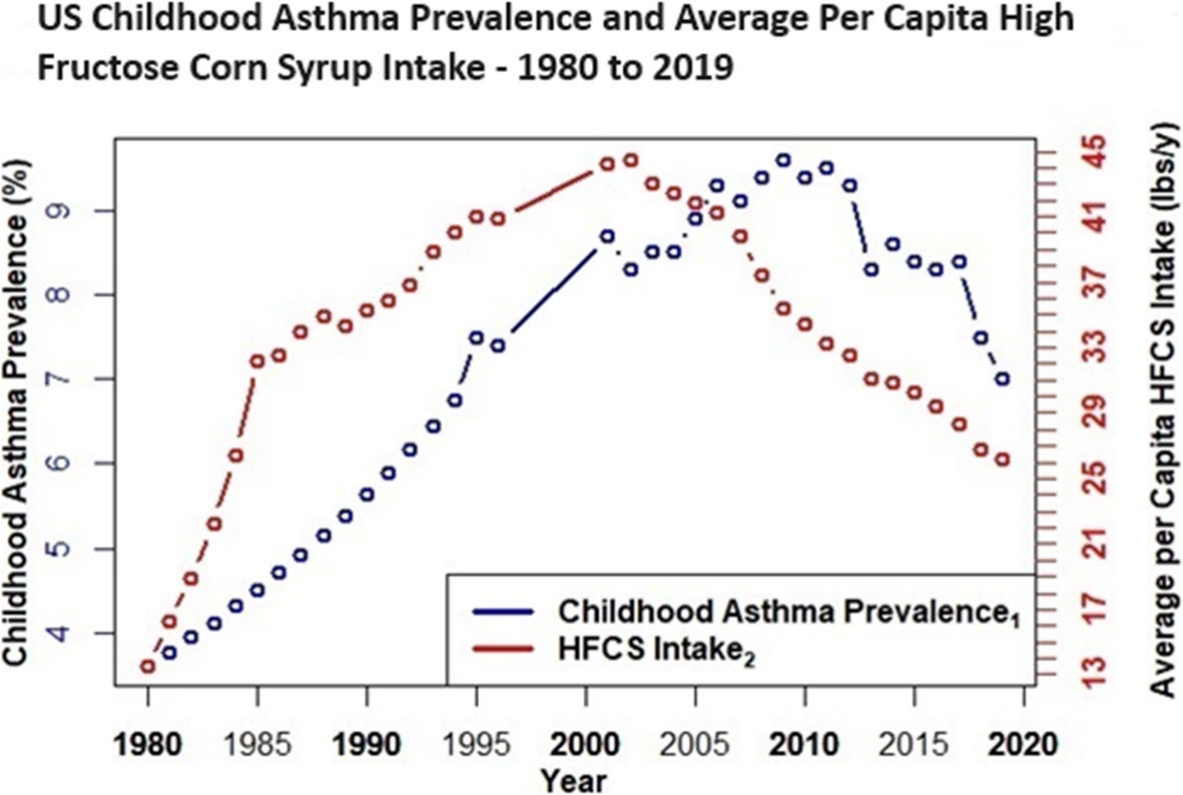
US Childhood Asthma Prevalence and Average Per Capita High Fructose Corn Syrup Intake – 1980 to 2019

**Fig. 7:**
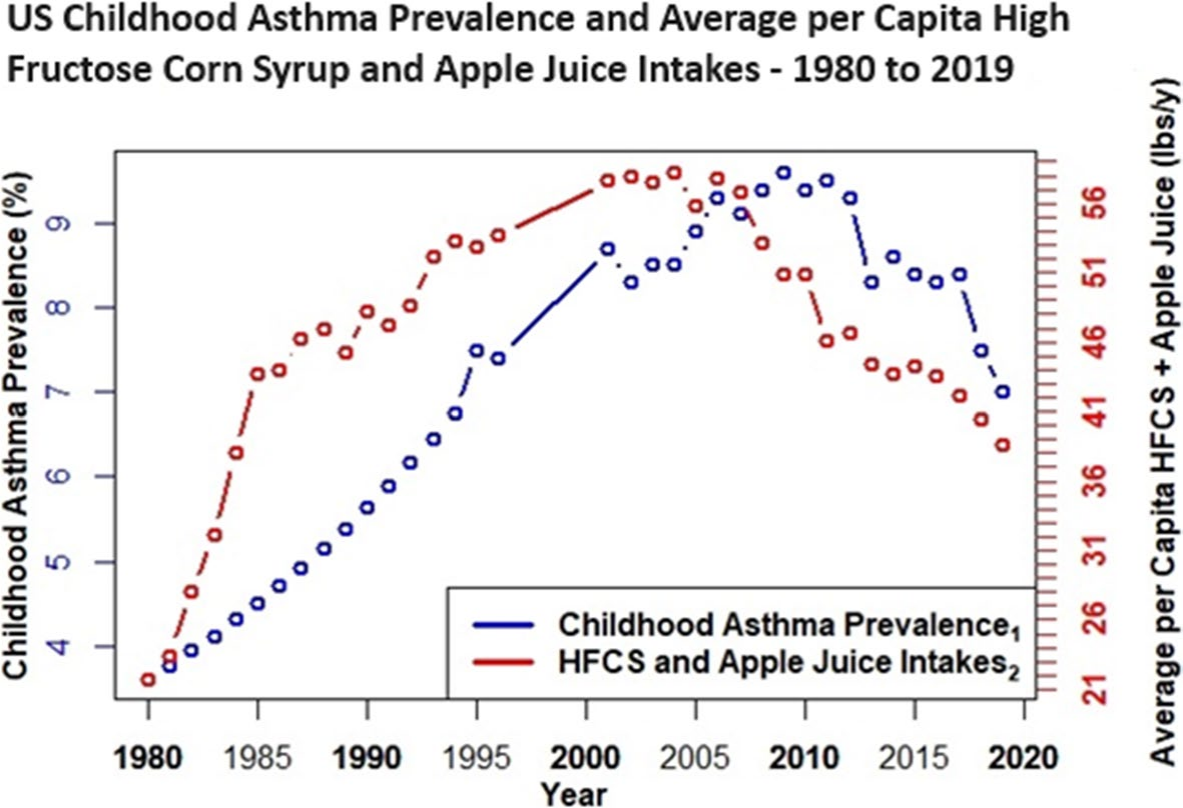
US Childhood Asthma Prevalence and Average per Capita High Fructose Corn Syrup and Apple Juice Intake – 1980 to 2019

Figures 4, 5, 6 and 7 depict combined plots of childhood asthma prevalence and average per capita intake trends for apple juice, orange juice, HFCS, and HFCS + apple juice (1980 – 2019). Orange juice intakes are based upon USDA Loss Adjusted Food Availability data, which include retail (6%) and consumer level loss allowances (10%). Childhood asthma prevalence data, as reported by the US Centers for Disease Control, are based upon self (parent) reported responses obtained from US National Health Interview Surveys for the years 1980–1996, 2001–2005 [73], 2006 – 2018 [74], and 2019 [75]. Childhood asthma prevalence from 1980 to 1996 reflects children with asthma anytime during the past 12 months, and although prevalence data were not available from 1997 to 2000, analyses suggest it remained fairly level during these 4 years [73–75]. Childhood asthma prevalence after 2000 is based on affirmative responses to the following questions: “Has a doctor or other health professional ever told you that your child had asthma?” and “Does your child still have asthma?” [73–75] See Figs. 4, 5, 6 and 7.

## Results

It is evident, from Figs. 1, 2 and 3, that the ubiquitous presence of HFCS in the US food supply has exposed consumers to more excess-free-fructose than at any other time in US history; that average per capita excess-free-fructose daily intakes from HFCS, at higher fructose-to-glucose ratios than are considered safe, exceeded dosages associated with fructose malabsorption (~ 5 g) beginning in the early 1980’s, as based upon estimates with a ≤ 20% consumer level loss factor (Figs. 1, 2 and 3 and Tables 1, 2 and 3); and although HFCS intake has dropped from its peak in 1999, average per capita excess-free-fructose daily dosages, from HFCS, continue to **exceed** pediatric (~ 5 g), and approach adult dosages (~ 10 g) associated with fructose malabsorption, and its health consequences. Daily intakes are understated because this analysis does not include unpaired fructose from agave syrup and crystalline fructose – high excess-free-fructose sweeteners. It is also evident that differences in HFCS fructose-to-glucose ratios and consumer level loss factors have a material effect on average per capita excess-free-fructose intake estimates (Figs. 1, 2 and 3 and Tables 1, 2 and 3).

Excess-free-fructose daily intake for individuals consuming HFCS with an average 1.5:1 fructose-to-glucose ratio, ranged from 0.10 g/d in 1970, to 11.3 g/d in 1999, to 6.5 g/d in 2019, and for individuals consuming HFCS with an average 1.9:1 ratio, intakes ranged from 0.13 g/d in 1970, to 16.9 g/d at peak consumption (1999), to 9.7 g/d in 2019, based upon estimates that included a 20% consumer level loss allowance. Importantly, 10% of adults are fructose malabsorption positive after a 12 g excess-free-fructose dosage, and contributions to daily dosages from crystalline fructose and agave syrup—sweeteners with high concentrations of unpaired fructose—are not included in this analysis, nor are contributions from apple juice.

Contributions to daily excess-free-fructose intake, from average per capita loss-adjusted apple juice intake, rose steadily from ~ 0.2 g/d (1970), to ~ 0.5 g/d (1984), to ~ 0.7 g/d at peak consumption (2007), and back down to ~ 0.5 g/d in 2019 (Table 4). By the early 1980’s, the tripling of apple juice intake, had added about a half a gram to average per capita excess-free-fructose daily intake (Fig. 1, plots D-F).

**Table 4:** Excess-free-fructose intake (g/d) from apple juice, as extrapolated from average per capita loss adjusted food availability (LAFA) data^b^

**Year**	**Unadjusted per capita availability of apple juice in lb/yr**^b^	**Per capita availability of apple juice (lb/y) after loss from primary to retail (26.7%)**^b^	**Per capita availability of apple juice (lb/y) after 6% retail loss allowance**^b^	**Per capita availability of apple juice (lb/y) after 10% consumer level loss allowance**^b^	Per capita availability of apple juice (g/d) after 10% consumer level loss allowance—a g/d = ((lbs/yr * 453.59)/365)	**Per capita Fructose fraction in apple juice in g/d**	**Per capita Glucose fraction in apple juice in g/d**	**Average per capita Excess-free-fructose intake from apple juice (g/d)**
1970	6.44	4.72	4.4	4.00	4.97	0.32	0.15	0.17
1971	7.10	5.21	4.9	4.41	5.48	0.34	0.16	0.18
1972	5.49	4.03	3.8	3.41	4.23	0.27	0.12	0.14
1973	4.67	3.43	3.2	2.90	3.60	0.23	0.11	0.12
1974	5.97	4.38	4.1	3.70	4.60	0.28	0.13	0.15
1975	6.94	5.09	4.8	4.31	5.35	0.34	0.15	0.18
1976	6.36	4.66	4.4	3.94	4.90	0.31	0.14	0.17
1977	7.96	5.84	5.5	4.94	6.13	0.39	0.18	0.21
1978	9.67	7.09	6.7	6.00	7.46	0.47	0.22	0.25
1979	10.76	7.89	7.4	6.67	8.29	0.52	0.24	0.28
1980	13.15	9.64	9.1	8.16	10.14	0.64	0.29	0.34
1981	11.64	8.53	8.0	7.22	8.97	0.56	0.26	0.30
1982	14.71	10.79	10.1	9.13	11.34	0.71	0.33	0.38
1983	15.97	11.71	11.0	9.91	12.31	0.77	0.36	0.42
1984	18.57	13.62	12.8	11.52	14.32	0.90	0.41	0.48
1985	18.57	13.62	12.8	11.52	14.32	0.90	0.41	0.48
1986	18.32	13.43	12.6	11.37	14.12	0.89	0.41	0.48
1987	19.61	14.38	13.5	12.17	15.12	0.95	0.44	0.51
1988	19.32	14.17	13.3	11.99	14.90	0.94	0.43	0.50
1989	17.61	12.91	12.1	10.92	13.58	0.85	0.39	0.46
1990	20.93	15.35	14.4	12.98	16.13	1.01	0.47	0.55
1991	18.35	13.46	12.7	11.39	14.15	0.89	0.41	0.48
1992	18.96	13.90	13.1	11.76	14.62	0.92	0.42	0.50
1993	21.57	15.82	14.9	13.38	16.63	1.04	0.48	0.56
1994	21.51	15.77	14.8	13.34	16.58	1.04	0.48	0.56
1995	19.10	14.01	13.2	11.85	14.73	0.92	0.43	0.50
1996	20.56	15.08	14.2	12.76	15.85	1.00	0.46	0.54
1997	18.68	13.70	12.9	11.59	14.40	0.90	0.42	0.49
1998	21.75	15.95	15.0	13.50	16.77	1.05	0.49	0.57
1999	21.61	15.85	14.9	13.41	16.66	1.05	0.48	0.56
2000	21.59	15.84	14.9	13.40	16.65	1.05	0.48	0.56
2001	21.50	15.77	14.8	13.34	16.58	1.04	0.48	0.56
2002	21.66	15.88	14.9	13.44	16.70	1.05	0.48	0.57
2003	23.34	17.12	16.1	14.48	18.00	1.13	0.52	0.61
2004	25.52	18.71	17.6	15.83	19.67	1.24	0.57	0.67
2005	22.47	16.48	15.5	13.94	17.32	1.09	0.50	0.59
2006	26.63	19.53	18.4	16.52	20.53	1.29	0.59	0.70
2007	27.40	20.10	18.9	17.00	21.13	1.33	0.61	0.72
2008	25.27	18.53	17.4	15.68	19.48	1.22	0.56	0.66
2009	25.13	18.43	17.3	15.59	19.38	1.22	0.56	0.66
2010	26.56	19.47	18.3	16.48	20.47	1.29	0.59	0.69
2011	20.76	15.23	14.3	12.88	16.01	1.01	0.46	0.54
2012	22.66	16.62	15.6	14.06	17.47	1.10	0.51	0.59
2013	21.42	15.73	14.8	13.31	16.51	1.04	0.48	0.56
2014	20.58	15.19	14.3	12.85	15.87	1.00	0.46	0.54
2015	22.66	16.65	15.6	14.08	17.47	1.10	0.51	0.59
2016	22.63	16.72	15.7	14.14	17.44	1.10	0.50	0.59
2017	22.24	16.40	15.4	13.87	17.15	1.08	0.50	0.58
2018	22.14	16.20	15.2	13.71	17.04	1.07	0.48	0.59
2019	20.23	14.84	13.9	12.51	15.55	0.98	0.45	0.53

^a^There are 1.78 g of fructose and 0.82 g of glucose/ounce of apple juice or 5.73 g of fructose and 2.63 g of glucose/100 g of apple juice [29]

^b^U.S. Department of Agriculture, Economic Research Service. 2012. The ERS Food Availability (Per Capita) Data System. Economic Research Service Home Page, https://www.ers.usda.gov/data-products/food-availability-per-capita-data-system/

https://www.ers.usda.gov/data-products/food-availability-per-capita-data-system/food-availability-documentation/

By 1980, daily average per capita excess-free-fructose intake from HFCS, when combined with a half cup of apple juice (~ 3.8 g of excess-free-fructose), **exceeded dosages associated with pediatric fructose malabsorption** (~ 5 g for a toddler weighing 10 kg (22 lbs.), i.e. 0.5 g/kg of body weight), across all HFCS variants, including the HFCS variant that is generally-recognized-as-safe (HFCS 55/45) (Fig. 2, plots D-F and Table 4), irrespective of which consumer level loss allowance was used, including the subjective, retroactively applied increase to 34%**.**

Throughout the period for which data is available (1970 – 2019), excess-free-fructose dosages g/d/y, from average per capita HFCS intake, remained below the pediatric fructose-malabsorption-associated-dosage (~ 5 g/d), only for the HFCS variant that is generally-recognized-as-safe (55/45), and only when the consumer level loss allowance was 34%, as recommended by corn refiners (Figs. 1, 2 and 3). Dosages reached ~ 4.7 g/d at peak HFCS consumption (1999). Hence, the *retroactively applied* increase in the consumer level loss allowance (~ 2012) for HFCS, from 20 to 34%, had a material effect on excess-free-fructose daily dosage estimates. The retroactively applied (1970’s) increase to 34% (~ 2012) appears self-serving.

It is evident (Figs. 4, 5, 6 and 7) that increasing intakes of apple juice and HFCS, but not orange juice (Fig. 5), parallel increases in pediatric asthma prevalence, an age group with higher fructose malabsorption vulnerability / prevalence at lower excess-free-fructose intake than other groups.

## Discussion

Results herein show that the practice of adding more fructose to HFCS, than is generally-recognized-as-safe [56], has a compounding effect on excess-free-fructose dosages. It is evident that by the early 1980’s, daily average per capita excess-free-fructose contributions, from HFCS, exceeded dosages associated with pediatric fructose malabsorption (~ 5 g) [15, 16] and its broader health consequences [4–54]. For individuals consuming HFCS at average or above average consumption levels, that contained higher than generally-recognized-as-safe fructose-to-glucose ratios [56], this milestone was reached between 1980 – 1982, a period coincident with the advent of the “unexplained” US asthma epidemic [73–78], and with increases in heart/kidney disease mortality racial disparities [79–84], and that is before considering contributions from apple juice.

The ability to absorb excess-free-fructose (EFF), i.e., the type of fructose that occurs when the fructose-to-glucose ratio exceeds 1:1, is saturable and ranges widely from ~ 5 g to ~ 50 g [4–17]. EFF, but not sucrose or equimolar concentrations of fructose and glucose, is associated with fructose malabsorption [6], a condition which is often *unaccompanied* by gas and bloating [14]. Hence, the practice of adding more fructose to HFCS [1, 2], than is generally-recognized-as-safe [56], is a problem, particularly for unwitting fructose malabsorbers. The capacity to absorb excess-free-fructose is lower in children (~ 5 g) [15, 16] than adults (~ 10 g) [4–13], and limited research shows that African Americans have higher fructose malabsorption prevalence than Hispanics at comparable excess-free-fructose intakes [55]. Notably, both groups (children and African Americans) have been disproportionately affected by the “unexplained” US asthma epidemic [73–77]. Weight does not explain the “unexplained” epidemic as higher asthma prevalence occurred primarily among normal weight individuals [78]. Research by Brinkley et.al. showed that Black individuals have elevated AGE / FruAGE levels (Carboxymethyl-lysine (CML)), which may be a consequence of disproportionately higher fructose malabsorption prevalence among Black people. AGE / FruAGE bind receptors (RAGE) that are mediators of asthma [83].

Importantly, very few natural foods ((~ 4.3 g EFF/medium-sized apple) [3], pears (~ 5.9 g EFF/medium-sized pear) [3], watermelons (~ 2.8 g EFF (1 diced 8-oz cup) [3] and mangoes (~ 4.4 g EFF/mango)) contain more fructose than glucose, and when eaten as whole fruits, in moderation, excess-free-fructose dosages are less likely to trigger malabsorption. Orange juice (OJ), the most consumed juice, contains nominal amounts of excess-free-fructose (0.4 g per 250 ml), despite the fact that it contains fairly comparable total fructose (11 g per 250 ml) as apple juice (15.7 g per 250 ml) [3]. Total fructose concentration *is an inadequate measure* of a food’s ability to trigger fructose malabsorption. The simultaneous ingestion of glucose, as occurs naturally in orange juice, improves fructose absorption, and thereby prevents malabsorption [6]. Unlike HFCS (1.9:1 and 1.5:1) and apple juice (~ 2.1:1), orange juice contains a near 1:1 fructose-to-glucose ratio. Therefore, it *is not* associated with fructose malabsorption. This fact is reflected in epidemiological research wherein, unlike HFCS sweetened beverages and apple juice, orange juice intake *was not* associated with asthma [32, 33]. Rather, OJ intake appeared protective [32].

There are no known genetic mutations associated with fructose malabsorption [85] which would not be a problem if not for the advent and widespread use of sweeteners with high fructose-to-glucose ratios (HFCS [1, 2], crystalline fructose (100% fructose), agave syrup (70%-90% fructose) [68, 86] and apple powder [3]). Excess-free-fructose average per capita intake estimates herein are therefore understated, as these additional sources of excess-free-fructose (crystalline fructose, agave syrup, and apple powder) have not been accounted for in this analysis.

Notably, the excess-free-fructose content in a 12 oz. can of cola with 39 g of HFCS is 3.9 g (21.45 – 17.55 = 3.9 g) when the fructose/glucose percentages are 55/45, i.e. the GRAS 1.2:1 ratio. The dosage increases to 7.8 g (23.4 – 15.6 = 7.8 g) when the HFCS variant is 60/40 (i.e. the 1.5:1 ratio), and jumps to11.6 g, when the HFCS variant is 65/35 (~ 25.3 g – 13.7 g = 11.6 g), i.e. the 1.9:1 ratio. From a national nutrition and health policy perspective, recommendations to reduce sugar sweetened beverage (SSB) intake are inadequate to address unwitting exposures, as high fructose-to-glucose sweeteners (HFCS [1, 2], crystalline fructose, apple powder [3], apple juice [3], and agave syrup [68, 86]) are ubiquitous in the US food supply. The practice of adding more fructose to HFCS, than is generally-recognized-as-safe, as reported by the University of Southern California’s Keck School of Medicine [1, 2], is likely not limited to beverages.

Nutrition labels should provide details of total fructose content, and more importantly, of the excess-free-fructose content in foods and beverages. This is consistent with recommendations by researchers at the Keck School of Medicine [1, 2]. Warnings are warranted when foods contain excess-free-fructose. Not only do nutrition labels *not provide* information of the fructose-to-glucose ratio in added sweeteners or the excess-free-fructose / unpaired fructose content, even if they did, independent oversight is needed to ensure compliance with safety standards. It is noteworthy that 100% crystalline fructose is promoted as a low glycemic alternative to table sugar, and is available for purchase in US grocery stores, but is void of malabsorption warnings [87]. Recommendations to limit added sugar intake as a means of improving diet quality / health are inadequate and fail to provide the consumer protections that are needed. It is worth noting that prior to an undisclosed settlement agreement, between the US Sugar Association and US corn refiners, HFCS was heavily promoted as “just like sugar” [88, 89]. This messaging likely slowed research of excess-free-fructose induced consequences of fructose malabsorption beyond gas, bloating and interference with nutrient absorption—research which continues to lack momentum.

Fructose in the gut causes dysbiosis – a condition linked to asthma, COPD, rheumatoid arthritis, diabetes, eczema, inflammatory bowel disease (IBD) and syndrome (IBS), cardiovascular disease (CVD), non-alcohol associated fatty liver disease (NAFLD), chronic kidney disease (CKD) and mental health and cardiometabolic disorders [17–25]. Unabsorbed excess-free-fructose in the gut glycates (fructosylates) dietary peptides and incretins to form immunogens (FruAGE) with far reaching consequences [26–30]. Lastly, the parallel trends between increasing average per capita HFCS/apple juice intake and the “unexplained” childhood asthma epidemic [73–78] are striking and consistent with the large and growing body of epidemiological research [31–45].

Asthma is characterized by cough not associated with a cold or flu, narrowing of the airways, wheeze, dyspnea / difficulty breathing, airway mucus hypersecretion that leads to infection and inflammation by providing an environment for microbial growth [90–92]. Gut and lung dysbiosis [25] and gastroesophageal reflux disease (GERD) are comorbidities of asthma [92]. Dysbiosis and subsequent dysregulation of microbiota-related immunological processes affect the onset of the disease, its clinical characteristics, and responses to treatment [25]. Uncontrolled asthma contributes to increased absenteeism and reduced quality of life. Emergency symptoms include bluish color to the lips and face, rapid pulse, severe anxiety due to shortness of breath, difficulty speaking and confusion. Breathing can temporarily stop and can lead to heightened risk of death [90–92].

## Conclusion

The displacement of sucrose with HFCS, its ubiquitous presence in the US food supply, and the industry practice of adding more fructose to HFCS than is generally-recognized-as-safe, combined with the increased use of apple juice as a sweetener in foods and beverages, and growing use of crystalline fructose, agave syrup (70–90% fructose) and apple powder, have all contributed to unprecedented excess-free-fructose daily intake levels. Dosages have exceeded and continue to exceed levels that trigger fructose malabsorption (~ 5 g-10 g)—a condition with far reaching consequences. Excess-free-fructose promotes gut formation of asthma provoking, proinflammatory advanced glycation end-products (FruAGE) and causes gut dysbiosis – a disease associated with a growing list of chronic diseases including asthma, COPD, autoimmune disease, IBD, IBS, CVD, NAFLD, CKD, and cardiometabolic and mental health disorders.

## Data Availability

Not applicable.

## Abbreviations

CDC: United States Centers for Disease Control and Prevention
CKD: Chronic Kidney Disease
CVD: Cardiovascular Disease
CLL: Consumer Level Loss factors included in United States Department of Agriculture’s Loss Adjusted Food Availability data
COPD: Chronic Obstructive Pulmonary Disease
EFF: Excess-free-fructose; Fructose-to-glucose ratios that exceed ~ 1:1 ratio of fructose to glucose
HFCS: High Fructose Corn Syrup
IBD: Inflammatory Bowel Disease
IBS: Inflammatory Bowel Syndrome
LAFA: Loss Adjusted Food Availability data published by the United States Department of Agriculture
NAFLD: Non-Alcoholic Fatty Liver Disease
US: United States
USDA: United States Department of Agriculture
